# Supercapacitance and superinductance of TiN and NbTiN films in the vicinity of superconductor-to-insulator transition

**DOI:** 10.1038/s41598-021-95530-5

**Published:** 2021-08-10

**Authors:** A. Yu. Mironov, D. M. Silevitch, S. V. Postolova, M. V. Burdastyh, T. Proslier, T. I. Baturina, T. F. Rosenbaum, V. M. Vinokur

**Affiliations:** 1 Rzhanov Institute of Semiconductor Physics SB RAS, 13 Lavrentjev Avenue, Novosibirsk, Russia 630090; 2 Novosibirsk State University, Pirogova str. 2, Novosibirsk, Russia 630090; 3 Division of Physics, Mathematics, and Astronomy, California Institute of Technology, Pasadena, CA 91125 USA; 4 Institut de Recherches sur les lois Fundamentales de L’univers, Commissariat de L’énergie Atomique et aux Énergies Renouvelables-Saclay, Gif-sur-Yvette, France; 5 Terra Quantum AG, St. Gallerstrasse 16A, 9400 Rorschach, Switzerland; 6 Physics Department, City College of the City University of New York, 160 Convent Ave, New York, NY 10031 USA

**Keywords:** Physics, Condensed-matter physics, Phase transitions and critical phenomena

## Abstract

We investigate the low-temperature complex impedance of disordered insulating thin TiN and NbTiN films in the frequency region 400 Hz–1 MHz in close proximity to the superconductor–insulator transition (SIT). The frequency, temperature, and magnetic field dependencies of the real and imaginary parts of the impedance indicate that in full accord with the theoretical predictions and earlier observations, the films acquire self-induced electronic granularity and become effectively random arrays of superconducting granules coupled via Josephson links. Accordingly, the inductive component of the response is due to superconducting droplets, while the capacitive component results from the effective Josephson junctions capacitances. The impedance crosses over from capacitive to inductive behavior as films go across the transition.

## Introduction

As early as in 1978, Gerard ‘t Hooft in his brilliant work^1^ illustrated an absolute quark confinement as a dual analogy to absolute disappearance of the resistance in superconductors and termed the resulting extreme opposite to the superconducting state a “superinsulator.” A gauge theory of the Josephson junction arrays (JJA)^2^ predicted the ground state dual to superconductivity and coined independently the same name for this phase possessing an infinite resistance. Finally, the work^3^ independently rediscovered this phase arising as a result of the duality of the uncertainty principle for Cooper pairs, named it again a superinsulator, and reported an experimental evidence of a superinsulating state in TiN films. A comprehensive gauge theory of the superinsulating state^4^ rests on the gauge field theory that revealed the topological nature of the superconductor–insulator transition (SIT)^5^ and on generalization of the technique of^2^ onto finite temperatures. The superinsulating state, a mirror opposite twin of superconductivity, appears at the insulating side of the SIT^2,3^ and is an exemplary manifestation of the electric–magnetic duality^6^ extended onto a quantum realm.

The superinsulating properties, in particular, its infinite resistance, are an implication of the formation of the magnetic monopole Bose condensate^7^, a mirror twin of Cooper pairs condensate in superconductors, emerging at the insulating side of the SIT. Monopole Bose condensate squeezes electric field lines connecting the charges of the Cooper couple, i.e. a Cooper pair–anti-Cooper pair duo, into the Polyakov’s electric strings. Polyakovs strings confine the motion of charges making them immobile^4^, hence resulting in the infinite resistance. Small electric fields, $E<E_{\mathrm{c1}}$, where $E_{\mathrm{c1}}$ is a lower critical field, do not penetrate superinsulators. This can be viewed as an electric analogue of the Meissner effect; the corresponding dielectric permeability is zero, $\varepsilon = 0$^8^. If the separation between the constituent charges of a Cooper couple is small enough, the couple does not feel an immobilizing binding action of the connecting Polyakov’s string in an analogue to the asymptotic freedom phenomenon in particle physics. Therefore, in a sufficiently small superinsulating system the motion of charges is not impeded, and the system behaves like a metal.

At intermediate fields, $E_{\mathrm{c1}}<E<E_{\mathrm {th}}$, the Polyakov strings enter the superinsulator which, if large enough to accommodate the strained strings, shows an infinite resistance. Finally, at the threshold field $E=E_{\mathrm{th}}$, Polyakov strings break down and the superinsulator turns into a standard insulator demonstrating thermally activated resistance. These dc electric properties of superinsulators have been experimentally investigated in detail in^8^ and showed an excellent agreement with the theoretical predictions. At the same time, the ac properties of the superinsulating state, especially on approach to the SIT, remain unexplored.

To gain an insight into the expected ac behavior of the systems in the critical vicinity of the SIT, let us recall that in the critical vicinity of the SIT, the films acquire a self-induced granularity, i.e., becomes and array of superconducting granules connected by Josephson links^3^. Next, we note that because of this self-induced granular structure, the local superconducting gap remains finite even at the very transition (although the global superconductivity, of course, disappears). Hence such a system is pretty much like an array of capacitors^9^ connecting inductive elements, see Supplementary Information (SI). This implies that on approach to the SIT from the superconducting side, when the electron path length diverges, an increase in the inductance and a decrease in the capacitance of the system will be observed. This conclusion is consistent with the expected decrease in static dielectric permeability^8^ on approach to the SIT. Accordingly, further movement into the insulating side of the junction should increase the capacitance of the entire system.

Here we report the results of low-temperature measurements of the complex ac impedance in disordered insulating thin TiN and NbTiN films in a close proximity to the SIT. We demonstrate that as the applied magnetic field or change in the normal sheet resistance drive the films across the SIT, the character of the systems’ response crosses over from the highly inductive to highly capacitive behavior expected for the superconductor-to-superinsulator transition.

## Sample preparation

**Figure 1:**
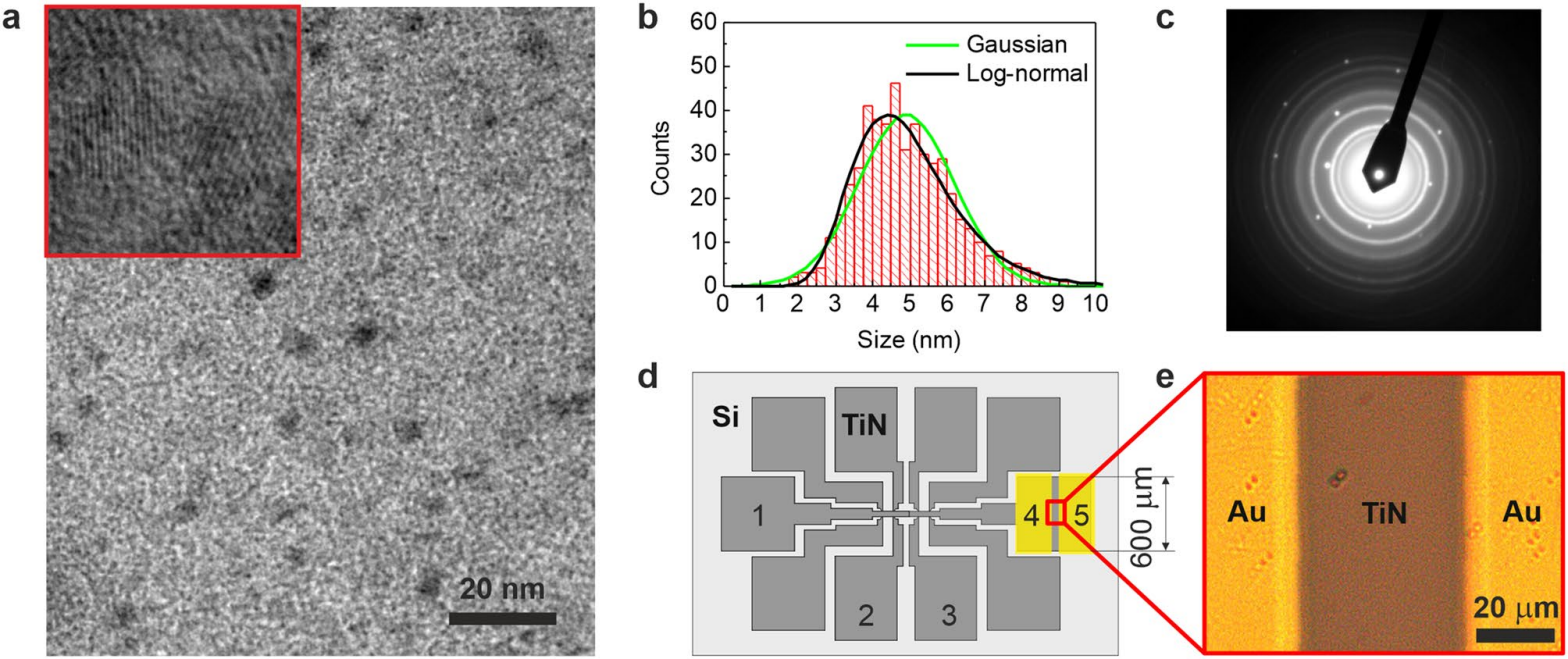
Experimental setup and film characteriization. (**a**) High resolution transmission electron micrograph of the plan view of the initial as-grown TiN film showing the polycrystalline structure, which is distinctly visible in the top-left corner inset in the image. The full size of the inset image is 20$\times$20 $\text {nm}^2$. (**b**) Histogram of the crystallite size distribution (the sampling number is 500). Note that the histogram is slightly asymmetric. The solid green and black curves represent the best fits by Gaussian and log-normal distribution functions, respectively. Gaussian distribution yields the average of the crystallite size of 4.9 nm with the standard deviation of 1.3 nm. Log-normal distribution produces 4.8 nm for the median crystalline size and 1.3 for geometric standard deviation. (**c**) Transmission electron-diffraction pattern of the film. The rings are characteristic to polycrystalline structures and correspond to fully stoichoimetric composition of the TiN film, while the point reflexes comprising a square array correspond to the crystalline lattice of the Si-substrate. (**d**) The layout of the sample prepared by the conventional UV lithography and plasma etching and displaying arrangement of the sputtered gold pads. The numbers mark contact pads. (**e**) Optical image of the part of TiN film (dark) with gold pads (yellow) between which the complex conductivity is measured.

The ac transport measurements are taken on the thin TiN and NbTiN films grown by the atomic layer deposition (ALD) technique as best technique for growing thin films^10,11^. The fabrication technique is described in detail in^3,12^. For growing TiN films we used $\text {TiCl}_4$, and $\text {NH}_3$ as gaseous reactants. To grow NbTiN films we used $\text {NbCl}_5$ additionally. For changing the stoichiometry of NbTiN films, we used а variation of the ratio of $\text {TiCl}_4$/$\text {NbCl}_5$ cycles during the growth^13^. The TiN films were deposited on the $\text {SiO}_2$ substrate. The superconducting properties of the ultrathin NbTiN films were optimized by utilizing the AlN buffer layers grown on top of the Si substrate^14^. The thickness of TiN films is $d=3.6$ nm and the thickness of the NbTiN films is $d=10$ nm. The film structure inspection by the high resolution transmission electron microscope (HRTEM) shows that the film is smooth, continuous, and uniform, and does not have pinholes. Figure 1a presenting a HRTEM image of the TiN film displays the polycrystalline structure with densely packed crystallites. The HRTEM image of NbTiN films display the same structure and are described in^12^. The crystallites are densely packed and they have different orientations. The boundaries between crystallites are atomically thin. The size distribution of crystallites together with the best fits by Gaussian and log-normal distributions giving average values 4.9 nm and 4.8 nm, respectively, is shown in Fig. 1b. Note the asymmetry of the histogram. The diffraction images of NbTiN films are similar to those of TiN films, see Fig. 1c, and are presented in^12^. The analysis of the diffraction data reveals that the both TiN and NbTiN crystallites have the same rock-salt crystal structure. Using the Vegard’s law, we find that the NbTiN film are solid solution of NbN and TiN taken in a ratio of approximately 7:3 for the sample U^12^, while samples N1, N2, N3 have NbN to TiN ratio approximately equal to 2:1. We patterned the TiN and NbTiN films into the configuration shown in Fig. 1d by standard UV lithography and plasma etching. Such a configuration allows for combined measurements of the dc resistance and ac complex impedance. Additionally on TiN mesastructure was manufactured of the 100 nm thick gold electrodes separated by the 50 $\mu$m by usual thermal evaporated technic (see Fig. 1e). For NbTiN samples we use the same mesastructure without gold electrodes, but measurements were taken only between the contacts 1 and 5–6. The distance between the contacts was 2.5 mm. Resistivity measurements were performed at sub-Kelvin temperatures in helium dilution refrigerators. In order to drive our TiN film with thickness 3.6 nm across the SIT we employ the controlled oxidation in the air at $T=600^{\circ }$ K which results in an increase of the sheet resistance at room temperature. Note that etching usually gives a more predictable result than oxidation. However, in the case of very thin films ($d < 4$ nm), the required etching time is less than 0.5 s, which significantly complicates the procedure for obtaining films close to the superconductor–insulator transition. In the case of NbTiN films, we used the plasma etching procedure for obtaining samples N1 and N2 from N3. The thicknesses of U and N1 samples are 10 nm, the thicknesses of N2 and N3 samples are indistinguishable and equal approximately to 9 nm.

## Results

**Figure 2:**
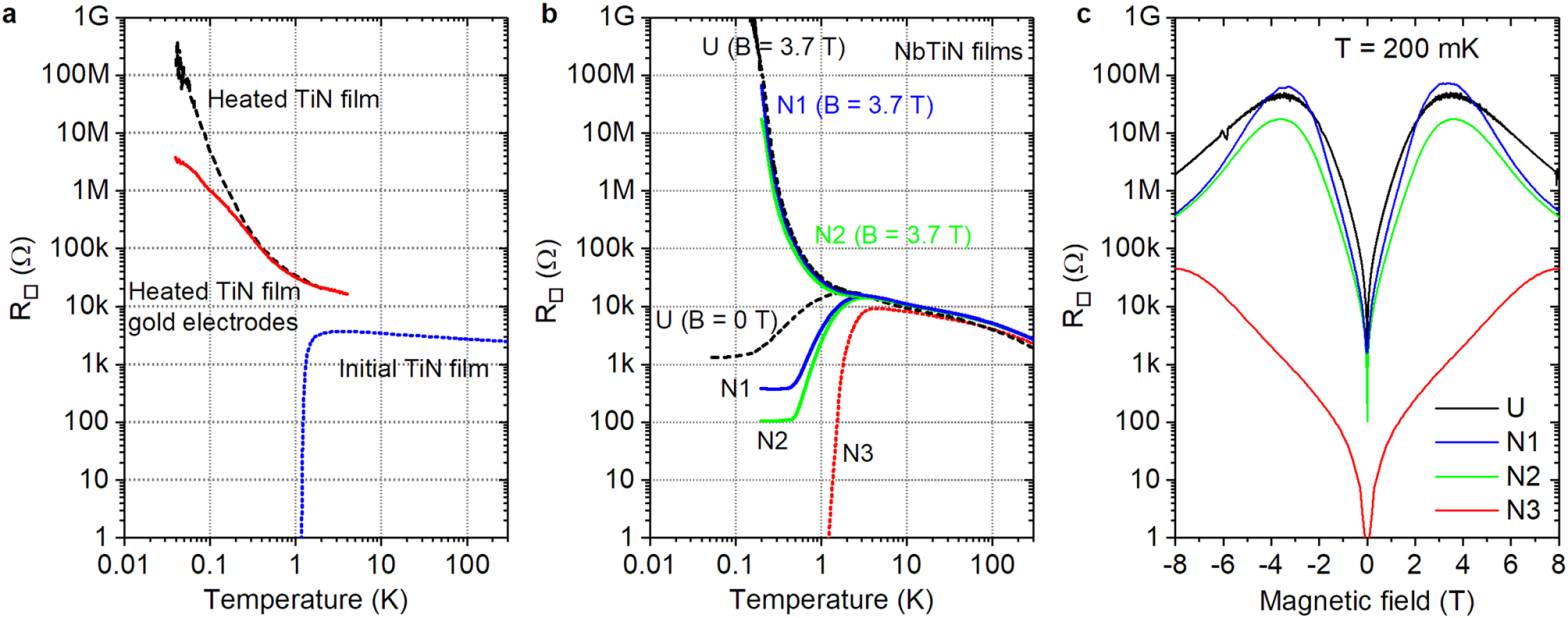
Superconductor–insulator transition. (**a**) Evolution of the representative TiN film from superconducting to insulating behavior. The initially superconducting films cross over to an insulator after heating to 600 K. After thermal evaporation of gold electrodes and decreasing distance between contacts from 2.5 mm to 50 $\mu$m, the insulating upturn slightly flattens. (**b**) The SIT in the NbTiN films. The changes of thickness (samples N1, N2, N3) and stoichiometry (samples UofC and N1) lead to changes of critical temperatures. The films are driven across the SIT by varying magnetic field. Note that superconducting samples saturate to metallic behavior at the lowest temperatures that correspond Bose-metallic behaviour^17^. (**c**) The magnetic field-induced SIT in NbTiN films for different samples at temperature of 200 mK.

Temperature dependencies of the resistance per square, $R_{{\square }}(T)$, are shown in Fig. 2 and plotted in a log–log scale spanning the window over four decades in temperature.

The evolution of $R_{{\square }}(T)$ for a representative TiN film across the superconductor-to-insulator transition is shown in Fig. 2a. The initial film is a superconductor. After heating, See Supplementary Information (SI), it becomes an insulator. The strength of the insulating behavior depends on the distance between electrodes. As demonstrated in^4^ superinsulating properties develop in full power only in the systems which are large enough to accommodate fully strained Polyakov’s electric strings confining the free motion of Cooper pairs. Therefore, decreasing the distance between electrodes, i.e., decreasing the system’s effective size, suppresses its superinsulating properties as has been indeed observed experimentally in NbTiN films^8,15^.

The NbTiN films exhibit a slightly different behaviour. Upon cooling down in the zero magnetic field, all NbTiN films undergo the superconducting transition which manifests via a severe resistance drop, see Fig. 2b. However, upon cooling, the resistance of all samples demonstrates the Bose-metallic saturation with the decreasing *T* well below the superconducting transition temperature, $T_{\mathrm c}$, which is determined by the inflection point of $R_{{\square }}(T)$. For the most disordered sample, this precedes the point at which the global phase coherence and, therefore, the zero resistivity set in^16^. Instead, with the decreasing film thickness (and increasing disorder), the spatial fluctuations of the superconducting gap become increasingly important, stimulating the formation of the self-induced texture of superconducting islands immersed into a non-superconducting environment^4,5^. The temperature $T_{\mathrm c}$ decreases with the decreasing film thickness and the consequent increase of the sheet resistance. In the magnetic field, in all the NbTiN films the magnetic field induced SIT is observed illustrated by the resistance growth in Fig. 2c. Moreover, samples U, N1 and N2 exhibit hyperactivated critical temperature dependencies below 300 mK.

**Figure 3:**
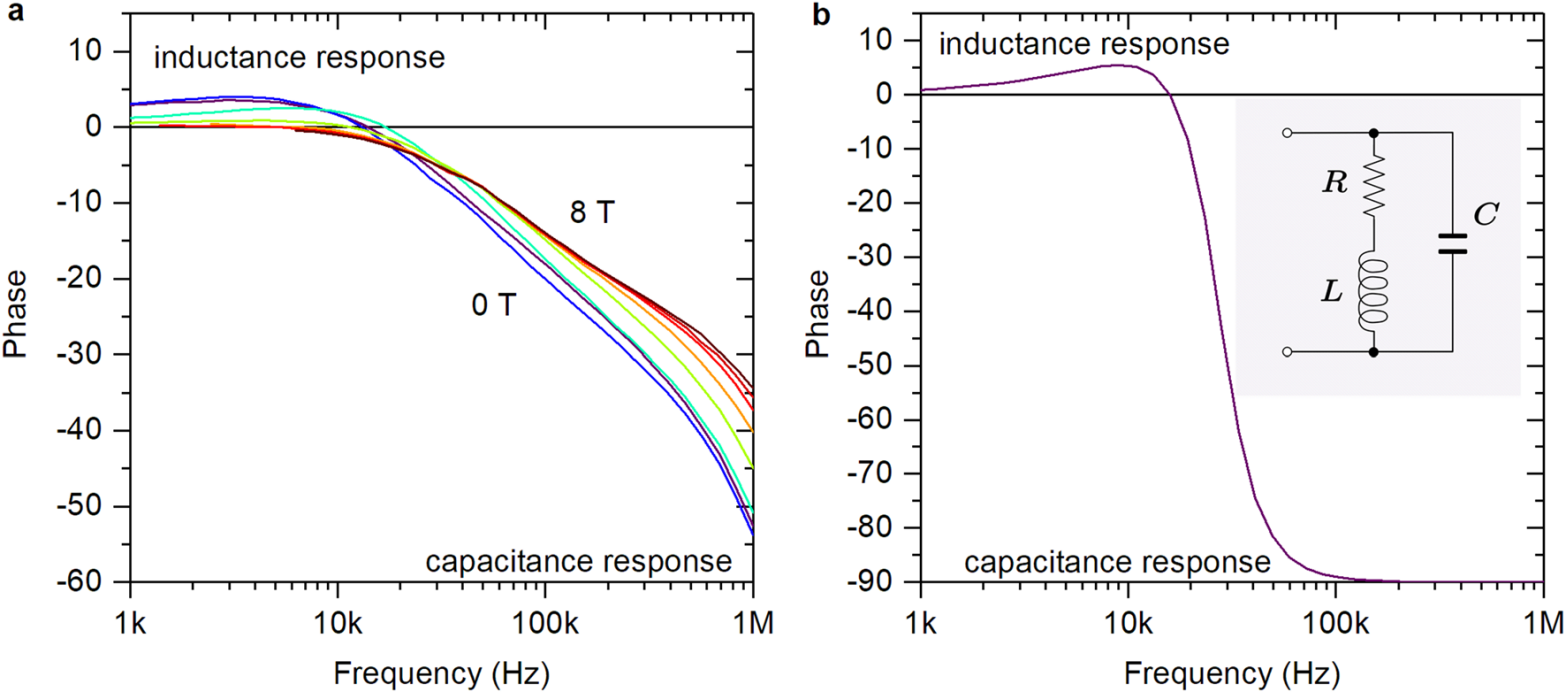
Frequency dependence of the complex impedance phase of the TiN film. (**a**) Results of the measurements performed between gold electrodes with the distance of 50 $\mu$m at T = 35 mK. We observed evolution from inductance to capacitance response with frequency increasing. (**b**) Typical frequency dependence of the $(RL)\Vert C$ circuit (see insert).

**Figure 4:**
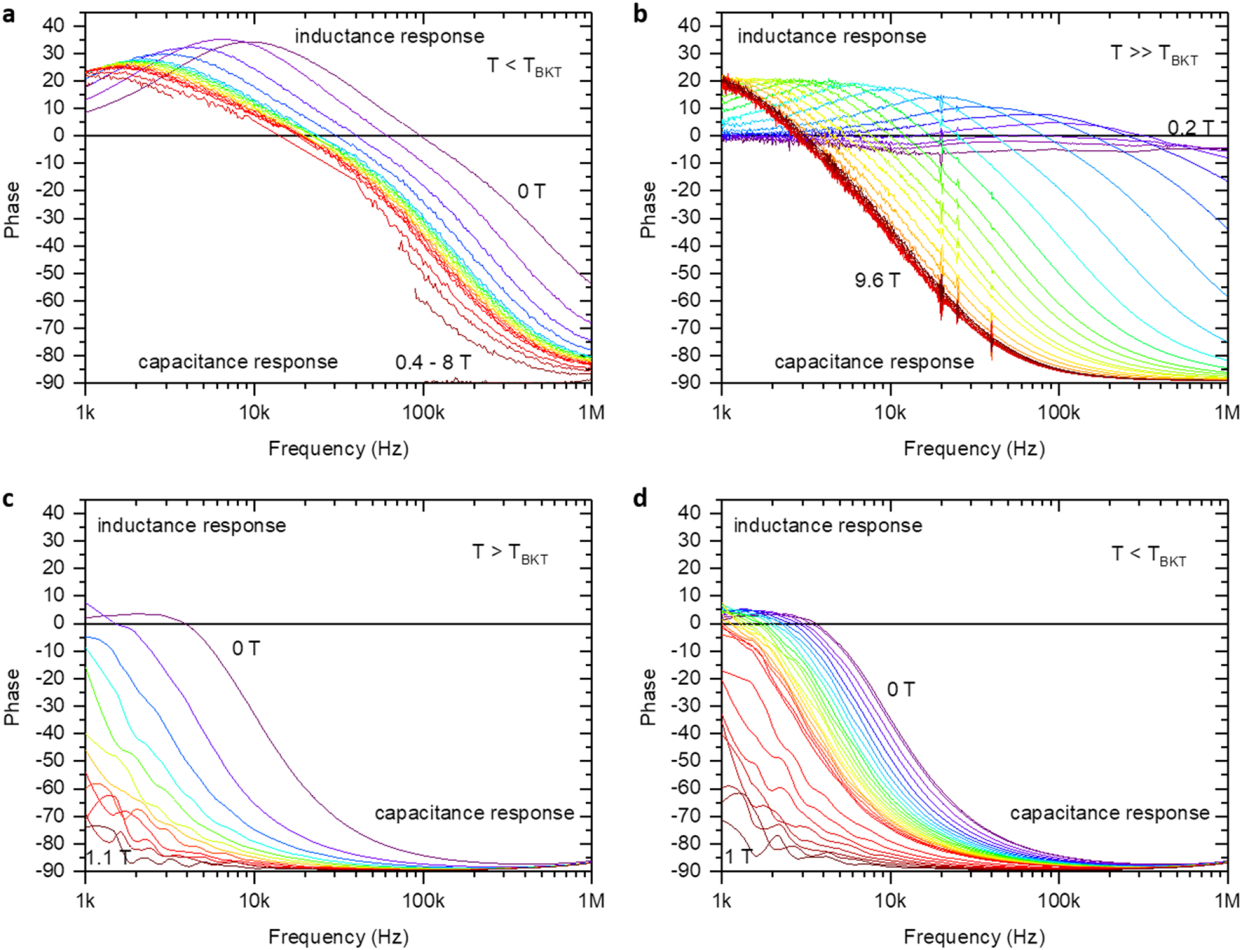
Frequency dependence of the phase of the complex impedance of the NbTiN films. Measurements were performed between contacts separated by the distance 2.5 mm. For all samples, we observed the evolution from inductive to capacitive response with increasing frequency. With increasing the magnetic field, the capacitive response dominates over the inductance response. (**a**) Sample U. Phase = 90 in magnetic field 0.4–8 T at temperature 35 mK. (**b**) Sample N1 at temperature 200 mK. (**c**) Sample N2. Phase = 90 in magnetic field 1.1–8 T at temperature 200 mK. (**d**) Sample N2. Phase = 90 in magnetic field 1–8 T at temperature 200 mK.

We investigate temperature, magnetic field, and frequency dependencies of the real and imaginary parts of the complex impedance both below (sample U) and above (TiN, N1–3 samples) the temperature of the charge Berezinskii–Kosterlitz–Thouless (CBKT) transition $T_{\mathrm{CBKT}}$^12^. Note that $T_{\mathrm{CBKT}}$ depends non-monotonically on the magnetic field with maxima in $T_{\mathrm{CBKT}}(B)$ for TiN sample being less than 30 mK and for NbTiN samples N1, N2, N3 that being less than 150 mK. For the U sample $T_{\mathrm{CBKT}}$ varies in the 35–180 mK interval^12^ so that the measurements on the U sample are taken at $T<T_{\mathrm{CBKT}}$. Representative results are shown in Fig. 3a for TiN film and in Fig. 4 for NbTiN films as frequency dependencies of the phase of the complex impedance in different magnetic fields. The obtained dependencies are non-monotonic functions of the frequency *f* and look quite similar to dependencies observed for the $(RL)\Vert C$ circuit shown in Fig. 3b. The positive values of phase correspond to domination of the inductance, the negative values of phase reflect the domination of the capacitive response. Note that an increase in the magnetic field leads to the suppression of the inductive response, which is associated with the sharp increase in the resistance in the magnetic field. One sees that the frequency-dependent phase of the complex impedance, assumes both positive and negative values. This indicates the presence of both capacitive and inductive components in the sample structure, see SI, and perfectly concurs with the presumed granular texture, i.e. being an array of superconducting islands in a insulating matrix connected by weak links, of the films involved, in full accordance with the description of a disordered film in the critical vicinity of the SIT^3–5^. At high frequencies, the response of the system is governed by the capacitances between the adjacent granules. Upon decreasing frequency, the inductive-resistance channel starts to dominate, so the phase of the impedance passes through the positive maximum and then tends to zero due to contribution from the randomly appearing resistive-inductive percolation paths through the granular array, see SI.

**Figure 5:**
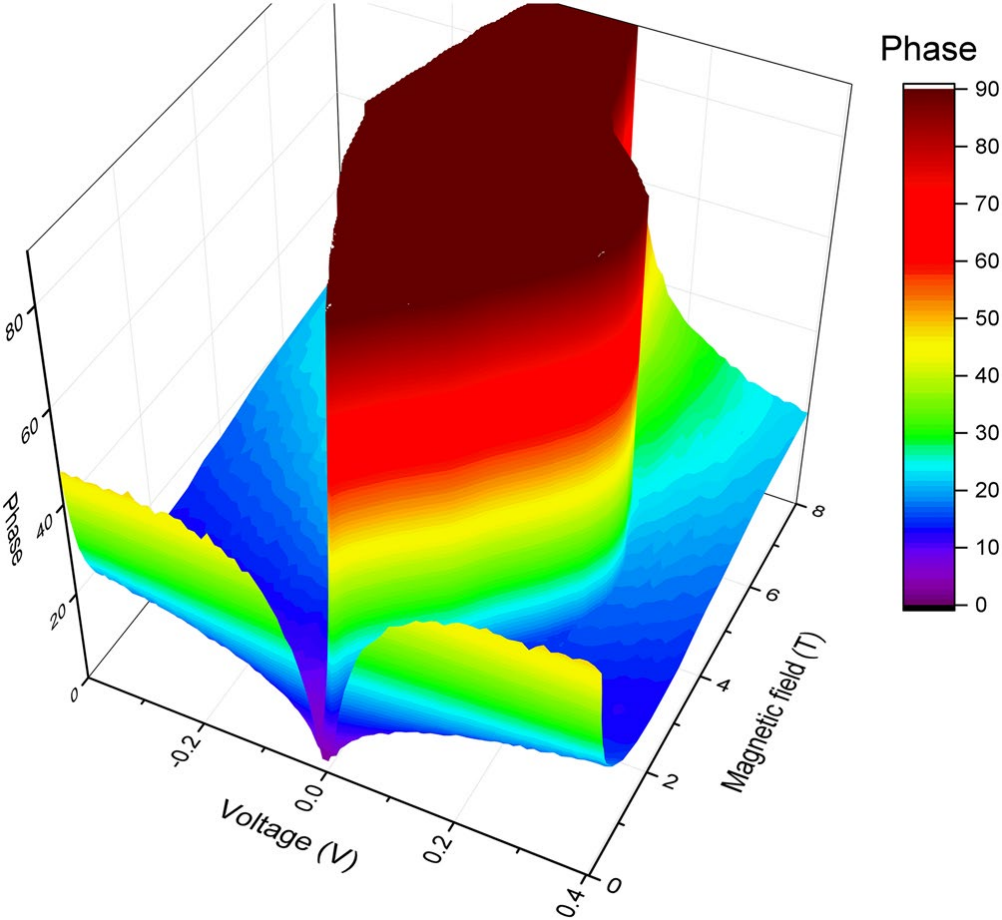
The 3D plot of bias voltage and magnetic field dependence of the phase of the sample U at 100 kHz at T = 35 mK. There is a threshold voltage where phase jumps from 90 degrees to 10–15 degrees. A similar threshold behavior is observed in the dc current–voltage dependences.

At $T<T_{\mathrm{CBKT}}$, the complex conductivity depends on the applied constant voltage, see Fig. 5. Moreover, we observe the threshold behavior both for real and imaginary parts of complex conductivity in the temperature range corresponding to the superinsulating state. The magnitude of the threshold voltage is the same for complex conductance as it is for the conductance measured at the constant voltage. This establishes that when the superinsulating state is destroyed, not only conductivity, but also the capacitance of the system, and hence its dielectric constant, change in a threshold manner.

## Data analysis

**Figure 6:**
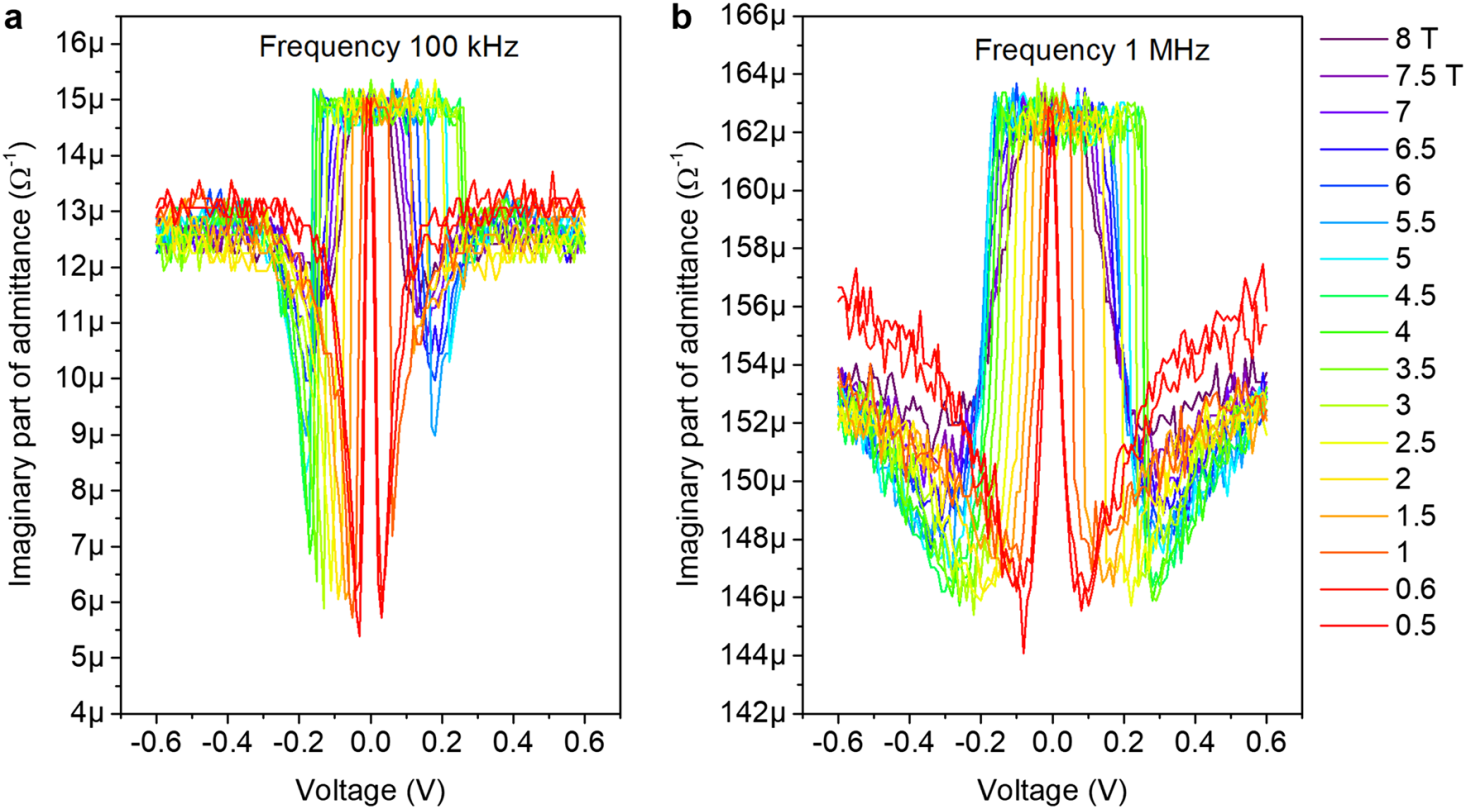
Bias voltage dependence of imaginary part of admittance of U sample in different magnetic fields at temperature 35 mK. Data were obtained by recalculation of |*Z*| and phase in Fig. 5. (**a**) Admittance at 100 KHz. The sharp threshold behavior is observed. (**b**) Admittance at 1 MHz. The threshold behavior is less pronounced than that at 100 kHz.

Figure 6 shows the dependences of the imaginary part of the admittance on the bias voltage at the frequencies of 100 kHz, 1 MHz and temperature 35 mK in various magnetic fields for the U sample. The plots exhibit the threshold-like behavior similar to that obtained by measurements at the constant voltage. Since the system is in a superinsulating state, we conclude that the imaginary part of the conductivity is related to the effective capacitance of the system, $Y = 2\pi f C$. At the same time, in addition to the capacitance of the sample itself, we inevitably have some contribution from the capacitance of the measuring circuit. In the first approximation, the characteristic capacitance of the sample can be estimated as the difference in capacitances at zero and at maximum voltages $V_{\mathrm{max}} = 0.6$ V. The simplest estimate, $C = (V_{\mathrm{max}}-V(0))/2\pi f$, gives $C = 3.7$ pF at $f = 100$ kHz and $C = 1.6$ pF at $f = 1$ MHz. Next, we estimate the dielectric constant of the system. To zeroth order, we assume that the sample is equivalent to a parallel-wire capacitor with the diameter of the wire $d = 10$ nm, the length of wire $w=50$ $\mu$m and the distance between wires $l = 2.5$ mm. In this case, the dielectric constant is $\varepsilon = C\ln ( l/2d + \sqrt{(l/2d)^2 -1})/\pi l\varepsilon _0$. After a simple calculation, we obtain $\varepsilon _{100k} = 3.5 \times 10^{4}$ at 100 kHz and $\varepsilon _{1M} = 1.5\times 10^{4}$ at 1 MHz. Such a large value implies that the electric field lines created by electrodes are practically all trapped within the film. This, in turn, suggests that the more adequate description of the capacitor may be the parallel plates approximation. This effective parallel-plate capacitor has plates of areas $S = 50$ $\mu$m x 10 nm with the distance between the plates of $l = 2.5$ mm. In this case, the dielectric constant is $\varepsilon = Cl/S\varepsilon _0$. This yields $\varepsilon _{100k} = 2.1\times 10^{9}$ at 100 kHz and $\varepsilon _{1M} = 0.9\times 10^{9}$ at 1 MHz. We thus conclude that the real value of the dielectric constant falls in between these limits $10^4 {-} 10^9$. Note that the dielectric constant decreases with the increasing frequency.

## Conclusion

**Figure 7:**
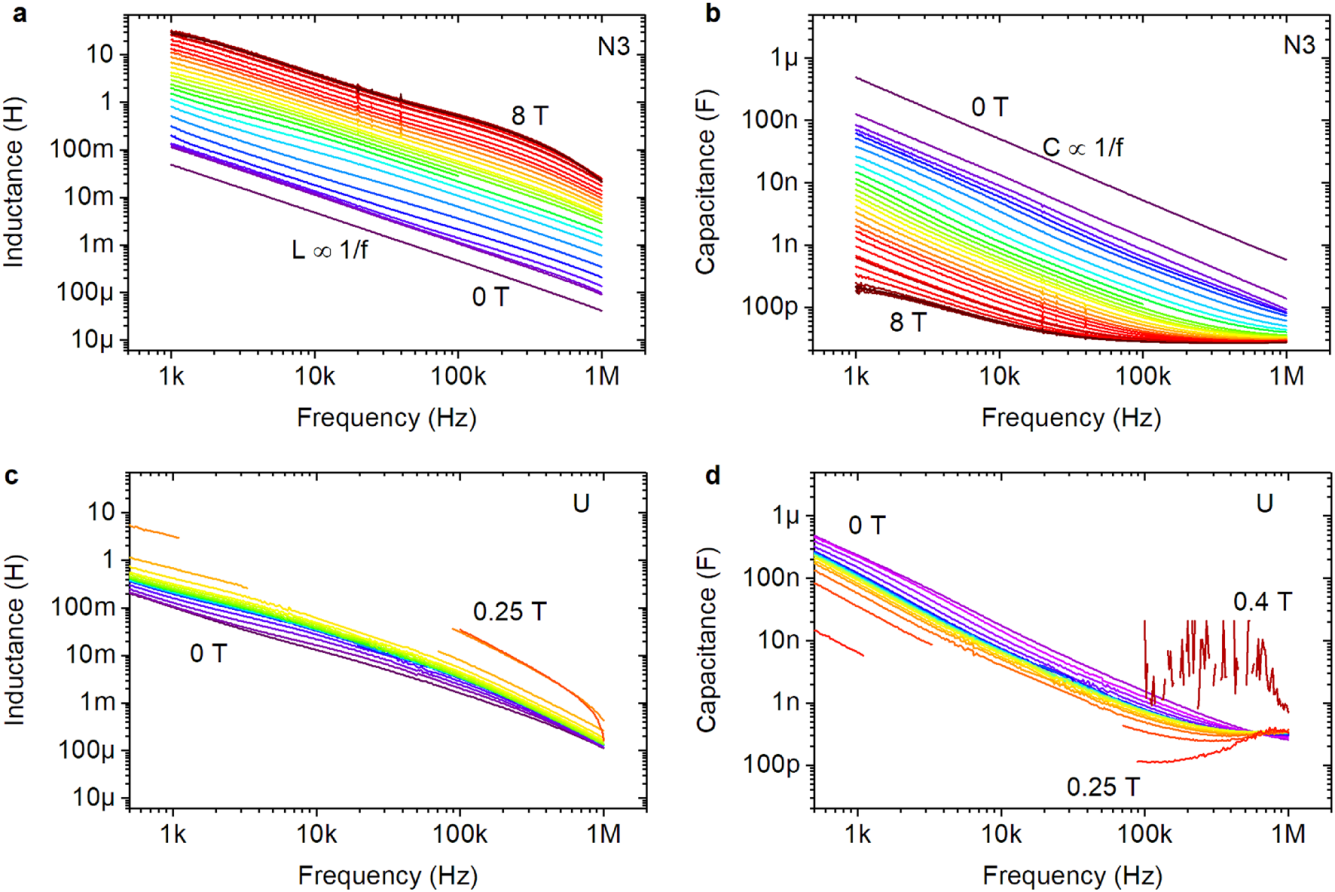
Frequency dependencies of the inductive and capacitive components of films complex impedance. (**a**) Frequency dependencies of the inductance of sample N3 at 0.2 K in different magnetic field. (**b**) Frequency dependencies of the capacitance of sample N3 at 0.2 K in different magnetic field. (**c**) Frequency dependencies of the inductance of sample U at 0.035 K in different magnetic field. (**d**) Frequency dependencies of the capacitance of sample U at 0.035 K in different magnetic field resulting from the analysis of the data presented in Fig. 4**a,b**.

The capacitance and inductance of the films are estimated in the framework of a simple model of the $(RL)\Vert C$-circuit. Representative results are shown in Fig. 7. We find that the inductance is inversely proportional to the frequency, $L\propto 1/f$, and has an incredibly huge magnitude. This observation is in a full concert with the expected large value of the kinetic sheet inductance $L_{\mathrm k}\approx \hbar R_{\mathrm{N}}/\pi \Delta$, near the SIT stemming from superconducting granules, where $R_{\mathrm{N}}$ is the sheet resistance of the normal state of the film just above superconducting transition temperature $T_{\mathrm c}$, and $\Delta$ is the magnitude of the superconducting gap in granules near the SIT. The obtained dependencies of the inductance and capacitance are non-monotonic functions of the magnetic field with the maxima of inductance and minima of capacitance at fields corresponding to the maxima of the magnetoresistance. This may result from the fact that in the critical region the percolation texture of the self-induced granularity reflects the change in the size and distance between the superconducting droplets under the action of the magnetic field. The details of the revealed behavior need further detailed investigation.

To summarize, the observed transition between the pronounced inductive and capacitive behaviors when films are driven across the SIT by magnetic field is in a full accord with the expected behavior of the system experiencing transition between superconducting and superinsulating states. The observed huge magnitudes of the inductance and capacitance are also in full agreement with the expected behavior in the vicinity of the SIT due to self-induced electronic granularity. Large $\varepsilon$ has already been observed in NbTiN films and was found to exhibit the behavior characteristic to percolation textures^12^. The large inductance follows from the fact that even in the closest proximity to the SIT at the superconducting side, the superconducting gap remains finite within the superconducting droplets ensuring a huge kinetic inductance. The critical question now is the study of the relaxation behavior of the system in the vicinity of the SIT which is crucial for both, understanding the fundamental frequency dependent properties of the topological phases near the SIT and possible applications of these materials. This will be discussed in the forthcoming publications.

## Methods

The growing of the films is based on the atomic layer deposition technique. The structure of films grown on the Si/$\text {SiO}_2$ substrate (TiN films) and the Si substrates with AlN buffer layers (NbTiN films) was investigated using a JEOL-4000EX electron microscope operated at 400 kV, with a point-to-point resolution of 0.16 nm and a line resolution of 0.1 nm. The details of the samples fabrication and structure are given in^3,12^.

To study the evolution of the low-frequency resistance of the films, we used the technique that has been tested many times^3,12,17^. The temperature *T* and magnetic field *B* dependencies of the resistance less 1 M$\Omega$ were measured by the standard four-probe low-frequency ac techniques at the frequency 1 Hz with the ac current 0.3 nA by the lock-in amplifier. So the current was sufficiently small to ensure the linear response regime as was verified by the direct measurements of the current–voltage (I–V) characteristics. The temperature *T* and the magnetic field *B* dependencies of the resistance higher 1 M$\Omega$ were measured using the standard two-probe low-frequency ac techniques at the frequency 1 Hz with the ac voltage 10 $\mu$V using the sensitive I–V-converter and lock-in amplifier.

The complex impedance between gold electrodes of TiN film and between contact 1–4,5 of U sample (see Fig. 1d) is measured as a function of frequency from 400 Hz and 1 MHz by LF Impedance Analyzer HP 4192A. The complex impedance contact 1–4,5 of N1,N2,N3 samples (see Fig. 1d) is measured as a function of frequency from 1 kHz and 1 MHz by Semiconductor Device Parameter Analyzer Keysight B1500A. The ac bias voltage is kept less than 10 mV. The magnetic field was applied perpendicular to the film surface. All measurements of N1, N2, N3 samples were performed in Oxford Dilution Refrigerator TLE200 in ISP SB RAS (Novosibirsk, Russia). All measurements of TiN film and U sample were performed in Oxford Dilution Refrigerator at the University of Chicago (Chicago, USA).

## Supplementary Information

Supplementary Information.

## Data Availability

The authors declare that all relevant data supporting the findings of this study are available within the article. Additional raw data, if necessary, are available upon request to corresponding author, VMV, vmvinokour@gmail.com.
