# Supplementary material for: Supercapacitance and superinductance of TiN and NbTiN films in the vicinity of superconductor-to-insulator transition
Source: Sci Rep. 2021 Aug 10;11:16181. doi: 10.1038/s41598-021-95530-5 (PMC8355108; doi:10.1038/s41598-021-95530-5)
Supplement: Supplementary file 1 — Supplementary Information. [file 41598_2021_95530_MOESM1_ESM.pdf]

Supplementary Information for: Supercapacitance and Superinductance of TiN and NbTiN films in the Vicinity of Superconductor-to-Insulator Transition

A. Yu. Mironov[1], D. M. Silevitch[3], S. V. Postolova[1], M. V. Burdastyh[1,2], T. Proslier[4], T. I. Baturina[1], T. F. Rosenbaum[3], and V. M. Vinokur[5,6,*]

[1]A. V. Rzhanov Institute of Semiconductor Physics SB RAS, 13 Lavrentjev Avenue, Novosibirsk 630090, Russia
[2]Novosibirsk State University, Pirogova str. 2, Novosibirsk 630090, Russia
[3]Division of Physics, Mathematics, and Astronomy, California Institute of Technology, Pasadena, CA 91125, USA
[4]Institut de Recherches sur les lois Fundamentales de L'univers, Commissariat de L'énergie Atomique et aux Énergies Renouvelables-Saclay, Gif-sur-Yvette, France
[5]Terra Quantum AG, St. Gallerstrasse 16A, CH-9400 Rorschach, Switzerland
[6]Physics Department, City College of the City University of New York, 160 Convent Ave, New York, NY 10031, USA
[*]Correspondence and requests for materials should be addressed to vmvinokour@gmail.com

ABSTRACT

We investigate the low temperature complex impedance of disordered insulating thin TiN and NbTiN films in the frequency region 400 Hz–1 MHz in close proximity to the superconductor-insulator transition (SIT). The frequency, temperature, and magnetic field dependencies of the real and imaginary parts of the impedance indicate that in a full accord with the theoretical predictions and earlier observations, the films acquire self-induced electronic granularity and can be viewed as random array of superconducting granules coupled via Josephson links. Accordingly, the inductive component of the response is due to superconducting droplets, while the capacitive component result from the effective Josephson junctions capacitances. The impedance crosses over from capacitive to inductive behavior as films are driven across the transition, characterized by the divergent dielectric response.

References

1. Vinokur, V. M. *et al.* Superinsulator and quantum synchronization. *Nature* **452**, 613 – 615 (2008).
2. Fistul, M. V., Vinokur, V. M., Baturina, T. I. Collective Cooper-Pair Transport in the Insulating State of Josephson-Junction Arrays. *Phys. Rev. Lett.* **100**, 086805 (2008).
3. Diamantini, M. C., Gammaitoni, L., Strunk, C., Postolova, S. V., Mironov, A. Yu., Trugenberger, C. A., Vinokur, V. M. Direct probe of the interior of an electric pion in a Cooper pair superinsulator. *Communications Physics* **3**, 142 (2020).

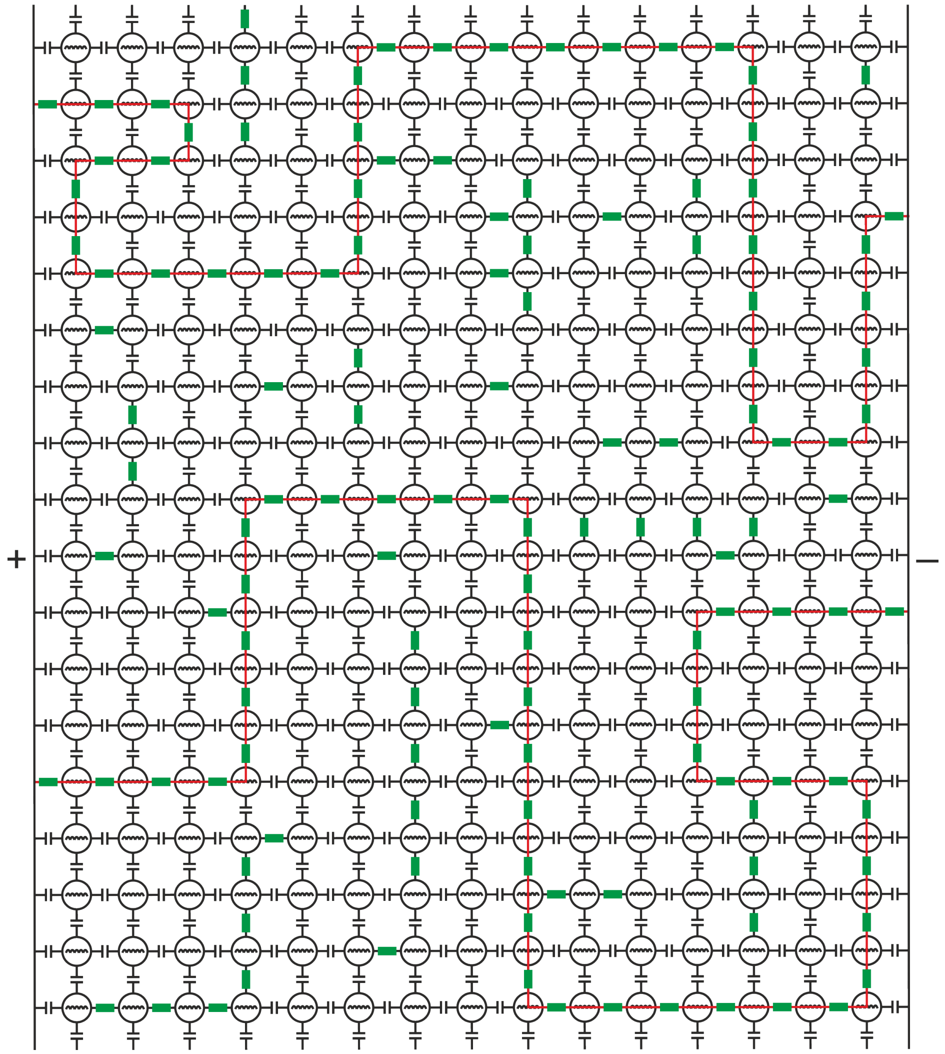

Figure 1. Schematic representation of the internal structure of a sample in the Bose-metal/insulating state, which assumes a self-induced granular electronic texture [1] and can be described as an array of superconducting granules connected by Josephson links. The circles represent these superconducting islands and provide an inductive response to applied ac electromagnetic field. Superconducting islands are connected by either capacitors or resistors (green rectangles) [2]. The red lines show exemplary direct current paths at voltages much less than the threshold voltage that causes a dielectric breakdown. The ratio of numbers of the resistive to capacitive links depends on the degree of disorder and/or magnetic field. When approaching the superconductor-insulator transition from the superconducting side, the number of the resistive contacts decreases, which leads to the decrease in the number of the possible electron paths and to increasing their lengths. Accordingly, this results in an increase in the inductance and in a decrease in the capacitance of the system. This conclusion is consistent with the expected decrease in static dielectric permeability [3] on approach to the SIT and agrees with our experimental observations. Accordingly, passing the SIT and moving further deeper into the insulating side of system should increase the entire system's total effective capacitance.
